# Effect of the Forming Zone Length on Helical Rolling Processes for Manufacturing Steel Balls

**DOI:** 10.3390/ma12182917

**Published:** 2019-09-09

**Authors:** Andrzej Gontarz, Janusz Tomczak, Zbigniew Pater, Tomasz Bulzak

**Affiliations:** Faculty of Mechanical Engineering, Lublin University of Technology, Nadbystrzycka 36, 20-618 Lublin, Poland (J.T.) (Z.P.) (T.B.)

**Keywords:** steel balls, helical rolling, helix tools

## Abstract

This paper begins with a brief overview of the methods for producing balls. It then discusses the rolling processes for producing balls in helical passes. Next, a method for designing tools for helical rolling (HR) is described. Six different cases of rolling using tools with helical passes of different lengths are modeled by the finite element method (FEM). The simulations are performed with the use of Simufact Forming version 13.3. Based on the 3D simulations, the distributions of effective strain, damage criterion, and temperature, as well as the variations in loads and torques, are determined. This study also predicts the rate and manner of wear of the helical tools, depending on the tool design. As a result, it has been found that an increased length of the helical forming passes is advantageous in terms of tool service life. It has also been found that excessive elongation of the forming zone is not cost-effective.

## 1. Introduction

The intensive development of global industry at the end of the 19th century led to increased demand for semi-finished products in the form of balls. Balls of steel are now extensively used in technology engineering, mainly as grinding and smashing media for ball mills and rolling elements for bearings. On a global scale, the annual demand for these semi-finished products amounts to millions of tons [1,2]. Given such huge demand, the production methods are continually improved and new production techniques that are less energy- and material-consuming must be developed.

**Figure 1:**
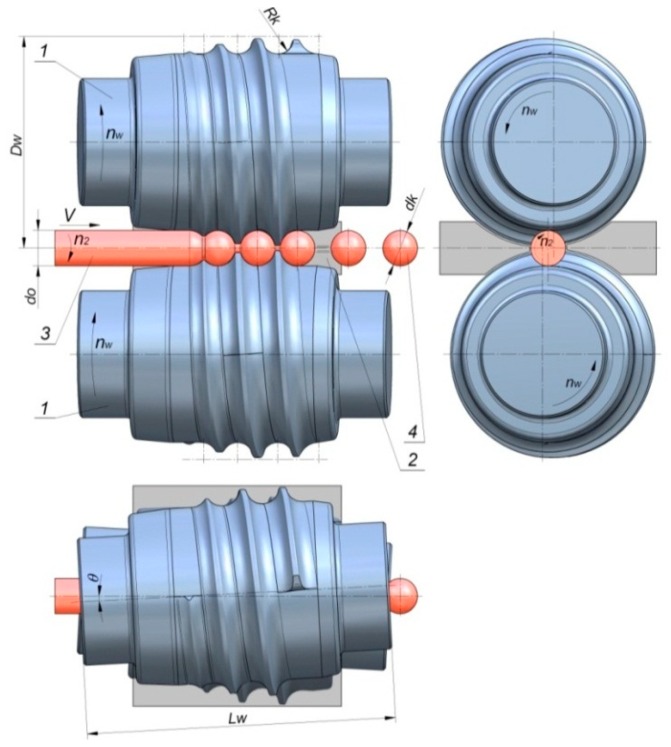
View of the helical rolling process for balls: 1—rolls with helix grooves, 2—guide plates, 3—workpiece, 4—manufactured ball.

There are numerous processes for producing balls, the most important being casting, die forging, and cross and skew rolling [3]. The effectiveness of these processes, as well as the strength and operational properties of the produced balls, depends, to a great extent, on the forming technique applied. The most popular ball production techniques include helical rolling (Figure 1), in which the workpiece in the form of a bar is formed between two identical rolls that have helical grooves on their surface. Compared to other processes, helical rolling offers numerous advantages, such as a high efficiency, reduced quantity of waste material, desired structure, lower energy consumption, and high product repeatability. 

The use of helical tools for manufacturing balls was first proposed in 1901 by Bornemann [4], who introduced rolls with parallel axes and helix grooves of varying form made on the surface of these rolls. The forming method developed by Bornemann was improved in 1928 by Hodge. He suggested that the rolling process be carried out using rolls with helical grooves and that the axes of these rolls be twisted by a few degrees [5]. To maintain the suitable position of the workpiece, linear guides were applied. In consequence of the skew position of the rolls, the rod workpiece would spontaneously be fed into the rolling zone during the process.

Although the fundamentals of the rolling process with helical tools for balls were predominantly designed in the United States, the method of rolling was first introduced on a massive scale in Russia. In the 1950s, an investigation team led by Celikov built the first Soviet ready-made skew mill for rolling and—based upon the machine’s design—developed a forming process by rolling for manufacturing balls with diameters ranging between 25 and 45 mm. In 1956, the OAO “EZTM” Company located in Elektrostal, a city in what is now Russia, started the manufacture of commercial mills for forming balls by rolling [6].

Despite their long history, helical rolling processes for fabricating balls have not been widely used in the metal industry. This mainly results from the difficulty in, first, designing the shape of the tool working surfaces such that they produce balls with the required parameters, and, then, constructing these tools. The low popularity of this process is reflected by the small number of publications on helical rolling. Moreover, the contemporary specialist literature offers practically no studies on the design of helical tools; the only studies available were published several dozen years ago in the former Soviet Union [7]. However, the solutions offered in these studies contain many marginalizations that were necessary then to design tools. Given the above, it can be claimed that rolling processes with helical tools for manufacturing balls have not yet been thoroughly investigated, and, thus, the current state of knowledge about these processes is far from exhaustive. For this reason, it is justified to undertake a study aimed at determining the effect of both technological parameters and tool geometry in helical rolling on the process stability and acceptable quality level of the produced balls.

## 2. Experimental Research

In Poland, rolling processes are not used in practice for ball manufacturing. Until recently, the only Polish contribution to the body of knowledge of helical rolling for balls was limited to the studies conducted by Dobrucki in the 1970s. Using a laboratory rolling mill, he conducted a series of tests of rolling balls made of commercially pure lead and steel. The results were published [8,9], yet they could not be considered satisfactory, because they very generally described the effect of the technological parameters of the rolling on the shape and surface defects of balls and crack formation.

**Figure 2:**
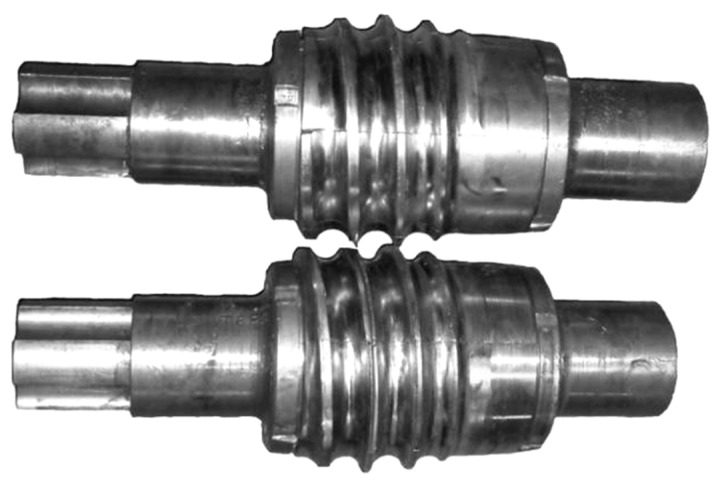
Kit of helical tool segments for forming 33 mm diameter balls.

Recently, broad studies were completed at the Lublin University of Technology to study rotary processes for fabricating balls made of scrap railway rails [10,11]. The research mainly focused on examining the use of cross wedge rolling to produce semi-finished balls. Given the numerous benefits of the helical rolling (HR) process, preliminary studies were conducted to investigate whether helical rolling processes could also be used for producing balls. A series of numerical simulations and experimental research of cross rolling balls with a diameter of 33 mm were performed as part of the research. Two helical tool kits for forming ∅ 33 mm balls were designed. The first kit (Figure 2) was designed in accordance with the outlines provided in the Russian specialist literature on the subject [6]. The other set of tools having a wedge-shaped cutting zone was designed according to a new sizing method worked out in Poland at the Lublin University of Technology [12].

**Figure 3:**
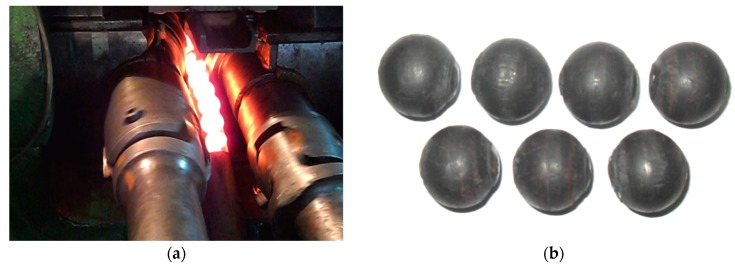
Balls manufactured by helical rolling in a tubular guide. (**a**) rolling process; (**b**) rolled balls.

Numerical simulations of the HR for balls with the designed tools were carried out by the finite element method (FEM), and the effects were then checked under laboratory conditions. Experimental tests of forming ∅ 33 mm balls by the helix tools were carried out using a two-roll rolling skew mill (Figure 3) that is available at the AGH University of Science and Technology in Cracow, Poland. This skew rolling mill is multi-faceted because it enables different sizing of the working tools. The rolling can be performed in two- and three-roll systems, using different guides: Disc guides, constant guides, rotary rollers (two-roll system), or a working roll (three-roll system).

**Figure 4:**
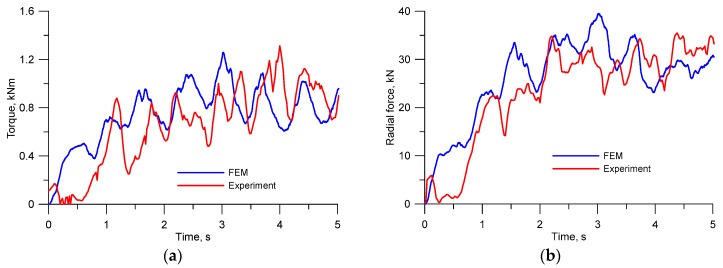
Force parameters in the helical rolling for producing ∅33 mm balls: (**a**) Torque; (**b**) radial force.

The research confirmed that skew rolling mills can be used to form balls, and FEM results showed good agreement with the experimental findings, particularly in quantitative terms (Figure 4). This proves that the FEM-based software is an effective tool for investigating this forming process. Given the above, a theoretical analysis was undertaken in this paper to investigate the influence of the basic parameters of helical rolling on the stability of the HR process for manufacturing grinding media.

## 3. Design of the Helical Tools for Rolling Balls

The helical method of rolling for manufacturing balls and the acceptable quality level of the obtained semi-products greatly depend on the tool design (the manner in which the helical tools are sized). The currently used methods for sizing helical rolls were evolved several dozen years ago in the Soviet Union, and they are all based on the establishment that the roll collar height is increased at a uniform rate during the forming process. Aiming to produce correctly shaped balls by helical rolling, the tools have to satisfy three circumstances subsequent to the metal stream pattern [7]:

- the quantity of the material limited in the roll (between two adjoining roll collars) should be supported invariable in the entire forming process;
- any variation in the roll collar contour and height should entail extension of the product;
- the material should be confined in the pass on relatively short lengths in order to prevent internal cracking.

**Figure 5:**
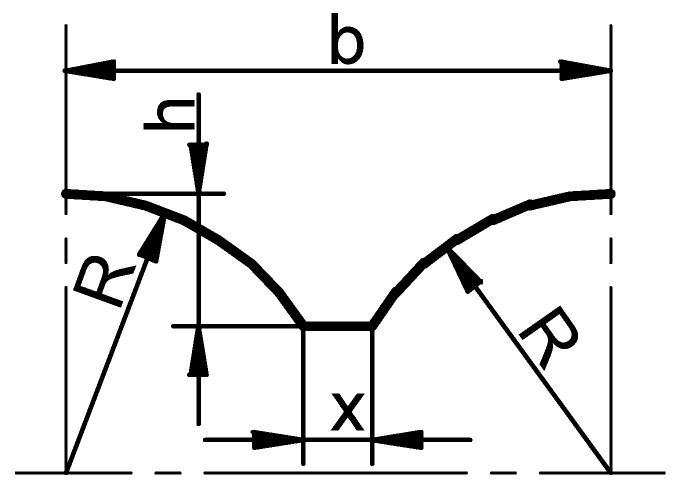
Profile of the roll collar used to calculate the tool geometry.

During HR, the roll impression is completely filled only if the material adjoins to the tool surface in a continuous mode. This condition is satisfied if the quantity of the metal extruded from the connector of the ball is equal to the increase in the quantity of the half-balls. According to Figure 5, the metal quantity V that is created by a single collar can be determined (at any time during the process) with this equation:

(1) $$V=\frac{2}{3}\pi \sqrt{R^{2}-{\left(R-h\right)}^{2}}\left[2R^{2}-{\left(R-h\right)}^{2}\right]+\pi x{\left(R-h\right)}^{2}$$

where: *R*—radius of ball, *x*—width of collar, *h*—height of collar, *b*—width of the roll pass.

In order to determine geometrical features of the roll collar (height h and width *x*), the quantity V of the metal deformed by a single collar has to be the same as the volume of the formed ball (including the connector of the ball), *V_K_* (*V = V_K_*). The width and height of the roll collars are determined based on the forming length defined by the rotation angle of the roll, *φ*, the value of which should exceed 450° (1 and ¼ revolutions of the roll) [6]. When designing the tool, it is usually adopted that the increase in the height of collar *h* is linear, which makes it possible to perform the rolling process at an invariable value of the deformation coefficient ∆*r*. The width of collar *x* is calculated as

(2) $$x=\frac{V_{K}-\frac{2}{3}\pi \sqrt{R^{2}-{\left(R-h\right)}^{2}}\left[2R^{2}-{\left(R-h\right)}^{2}\right]}{\pi {\left(R-h\right)}^{2}}$$

Additionally, to ensure the required tool strength, the width of the collar in the entire forming scope should be higher than the acceptable value *x_min_*:

(3) $$x_{\min}=1.7+0.04(R-10).$$

**Figure 6:**
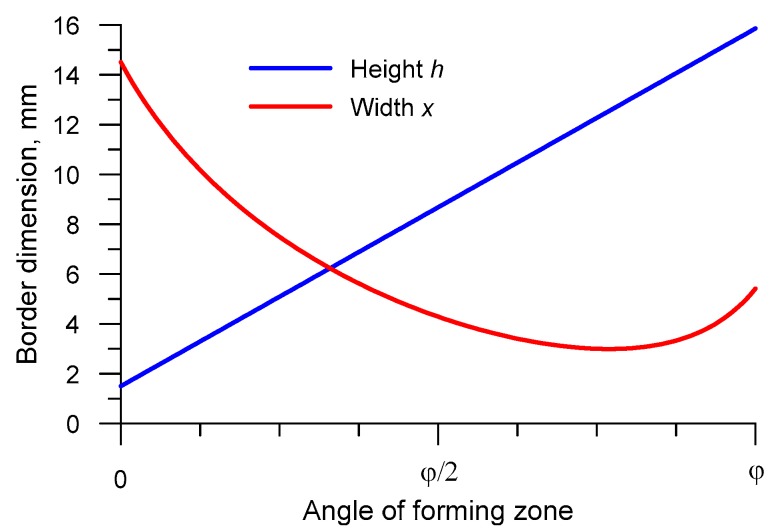
Roll collar parameters versus forming zone angle.

Taking the above into consideration, a family of tools for HR of ∅40 mm balls were designed. The rolls were then used in a numerical analysis of the process. Observed variations in the collar width *x* and height *h* (for the helical tools used to form ∅40 mm balls) are presented in Figure 6.

According to Figure 6, the collar width x is the maximum at the initial period of the rolling process. Therefore, it is at this stage when the highest forming forces can be expected to occur. In addition, with the increase in the width x, there is an increased probability of material cracking in the axial zone [7].

**Figure 7:**
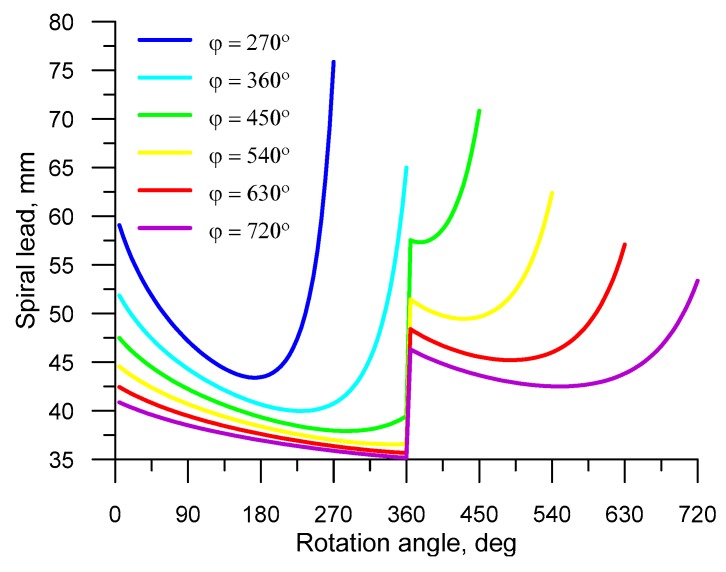
Helix pitch versus rotation angle *φ* in the forming zone (in helical rolling of 40 mm diameter balls).

The helical tools were sized such that the billet was shaped into balls over different forming lengths (at different rotation angles *φ*). In effect, the effect of the forming length on the helical rolling process, tool life, and acceptable product quality level could be determined. Six variants of tool sizing were applied for the forming lengths of 270°, 360°, 450°, 540°, 630°, and 720°. The forming length affects the helix pitch (Figure 7). Investigating the data in Figure 7, it can be noticed that all the designed tools have a forming collar coil on the tool with a varying pitch over the entire forming length. It can also be noticed that the helix pitch in the cutting zone first decreases and then increases. One can also observe that the shorter the forming length, the higher the helix pitch. The variations in the helix pitch on the roll collars are due to the metal flow pattern in the roll pass, and they result from the fact that the first two conditions (discussed in the third part of this paper), which are indispensable for the stability of the HR process, are satisfied.

**Figure 8:**
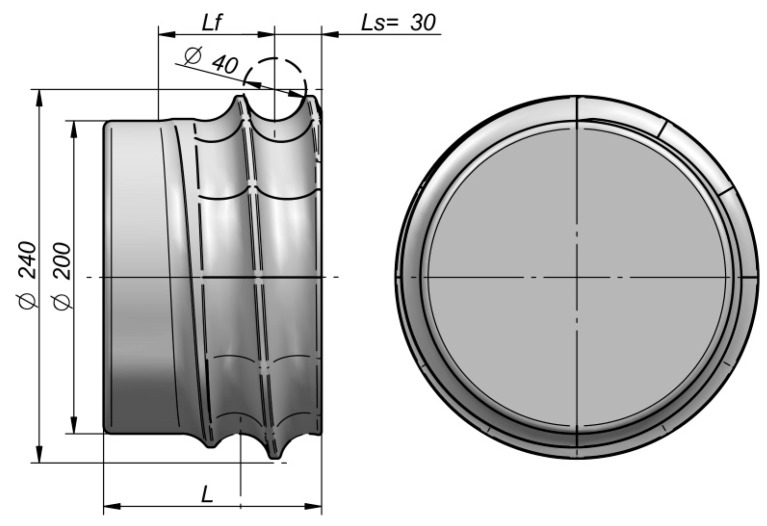
View of the helical tool (defined by *φ* = 360°) used in a numerical analysis, and its selected dimensions.

Table 1 lists the values of basic parameters of the designed helical tools for forming 40 mm diameter balls and the effect of the rotation angle, *φ*, on the forming length, *L_k_*, overall tool length, *L*, and working part length, *L_f_*. The tool with helical grooves and its basic dimensions are presented in Figure 8. As seen in the figure, with the decrease in the angle *φ*, the tool length increases, which leads to a higher tool wear. The cost of constructing tools with a longer forming length is higher, too. For this reason, it is fully justified to examine the impact of tool geometry on the rolling process with helical tools in order to determine an optimum range of the forming length that ensures the production of correctly shaped balls.

## 4. FEM Analysis

To estimate the impact of the forming length on the proposed HR process, product quality, and tool life, a series of numerical simulations were carried out by the FEM. The Simufact.Forming 13.3 software was used for computations [13,14].

**Figure 9:**
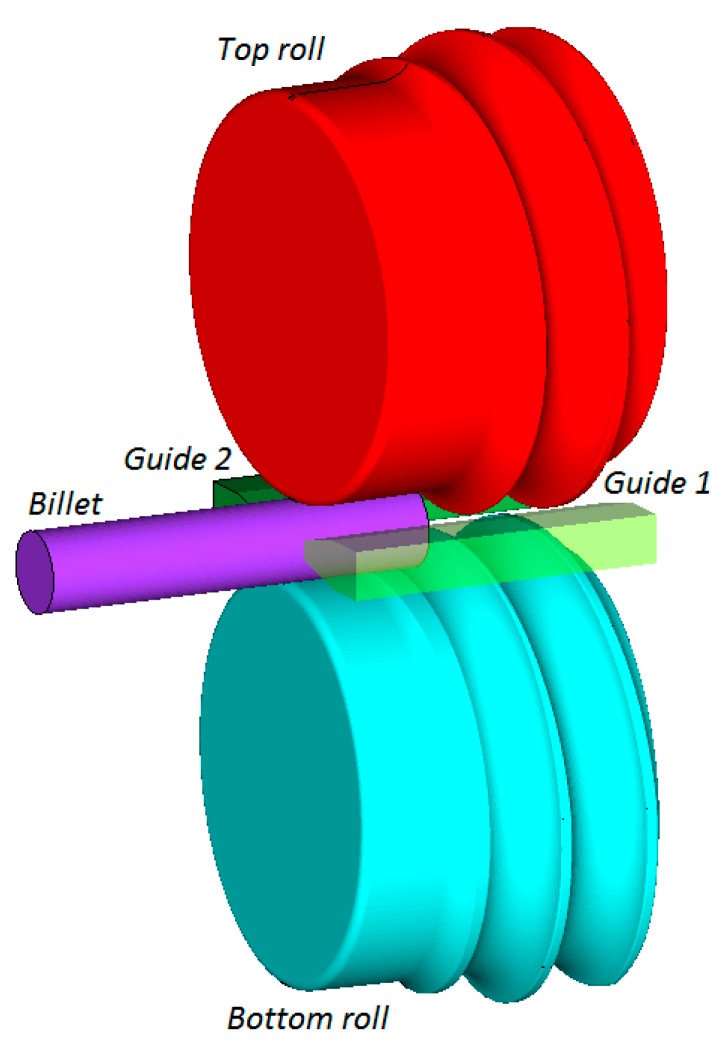
Virtual model of the analyzed rolling method for manufacturing ∅40 mm balls.

For the purpose of the FEM analysis, virtual models of the process for manufacturing balls were designed. The helix tools described in the previous section were used. One of these virtual models of the analyzed rolling method is shown in Figure 9.

Each virtual model consisted of two roll tools, two guide plates, and a workpiece in the form of a bar with a diameter of 40 mm. The discretization was performed using first-order eight-node mesh elements with a mean edge length of 1.5 mm. In addition, mesh refinement was performed in the areas of high strains (up to 0.75 mm). The billet material was modeled with the use of approx. 28,500 elements. To accelerate the computation process, the tools were assigned the properties of a rigid material while the billet material was featured by a rigid-elastic model. The working surfaces of the tools had helical grooves, and the radius of the roll collar was made exactly to the radius of the shaped ball. The billet was modeled to the material rheology of 100Cr6 steel. Supplied by the database material library of the Simufact software, this material model is characterized by the equation below [15]:

(4) $$\sigma_{p}=3570e^{-0.003612\cdot T}\cdot \varepsilon^{\left(0.0001992\cdot T-0.50004\right)}\cdot e^{\left(\frac{0.00004262\cdot T-0.11776}{\varepsilon }\right)}\cdot {\dot{\varepsilon }}^{\left(0.0002522\cdot T-0.09802\right)}$$

where *σ_p_* is the flow stress (MPa), *ε* is the effective strain, $\dot{\varepsilon }$ is the strain rate (1/s), and *T* is the temperature (°C).

The selection of this particular steel model was not random, as this steel grade is widely used for manufacturing balls for different bearings, among others [16]. 

The billet had a temperature of 1100 °C, and the temperature of the tools was 150 °C and was invariable during the process. The tools were rotated with the speed *n* of 60 rpm in the same sense. The surface contact of the pair billet-tool was modelled by the constant friction model. The friction factor *m* was set to 1 for the pair billet-rolls and to 0.4 for the pair billet-guides. The billet-tool heat exchange coefficient was set to 20 kW/m^2^·K and the billet-environment heat exchange coefficient was 0.35 kW/m^2^·K.

**Figure 10:**
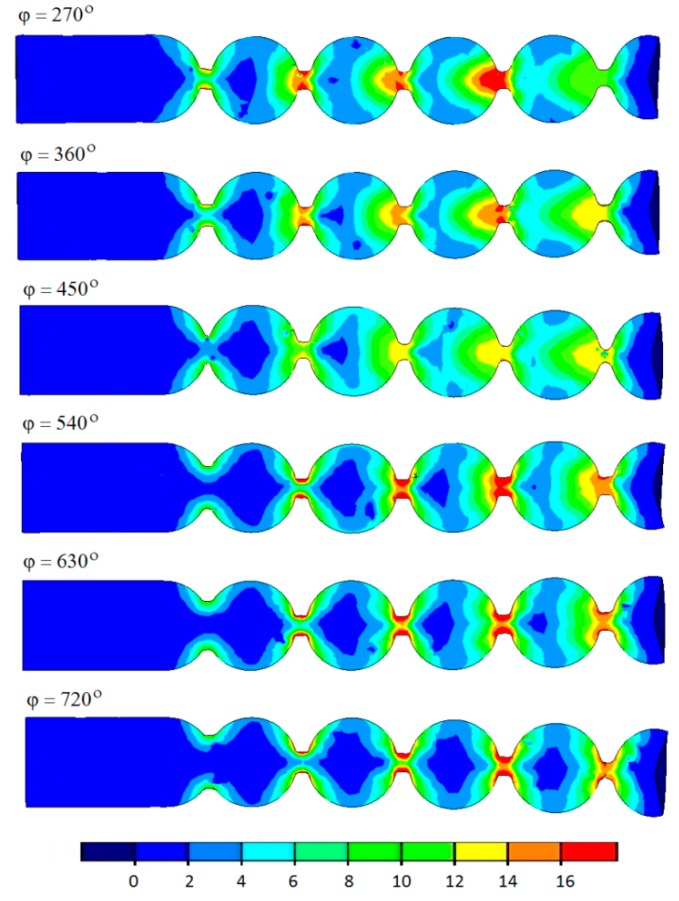
Effective strain in the axial section of semi-finished balls manufactured by helical rolling.

As a result of the simulations, the distribution maps of effective strains, temperatures, and the damage criterion, as well as changes in the rolling forces, were identified. The rate and pattern of tool wear were assessed, too. An analysis of the effective strains (Figure 10) reveals that the strains are not homogenous; they are distributed in layers, with the highest strains located near the necking.

In this region, the semi-finished product is deformed by the roll collars of increasing height, which causes a fast flow of the material in the radial and axial directions. Additionally, there occur considerable differences between the tangential velocities of the workpiece, which leads to a rapid circumferential metal flow, contributed by the effect of friction on the contact surface. The length of the roll collars also affects the extent of the area deformed during rolling. On increasing the forming length (higher angle *φ*), the strains become smaller and the area in which they occur is reduced.

**Figure 11:**
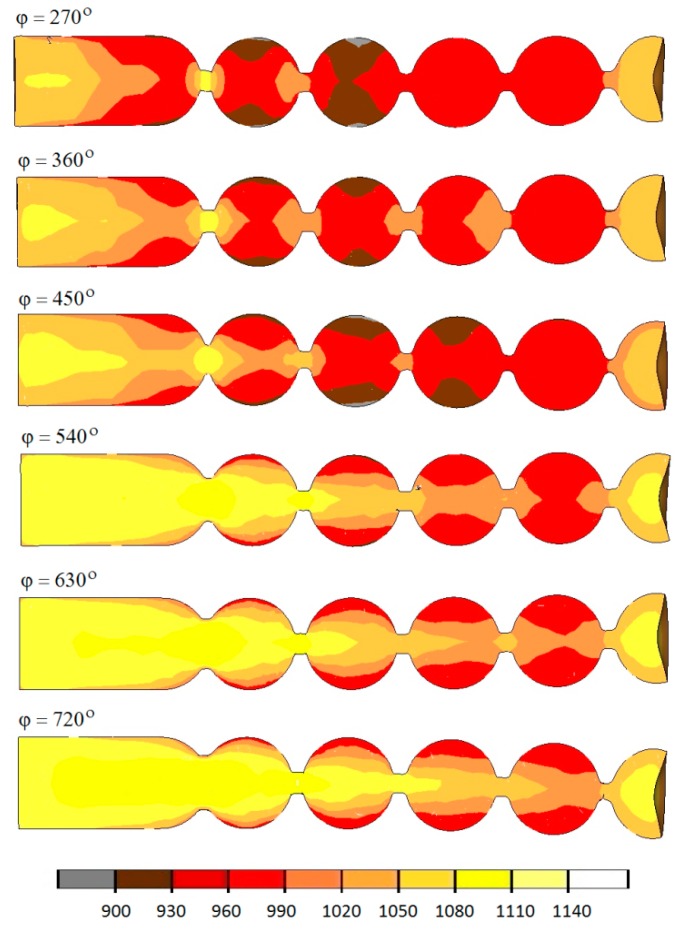
Temperature (in °C) in semi-finished balls produced by helical rolling.

Examining the numerical results of temperature (Figure 11) in the axial section of the manufactured balls, it can be seen that after 5 s, in all analyzed cases, the temperature is still high and stays in the hot forming conditions.

Interestingly, however, the decrease in the forming length leads to a decrease in the temperature of the produced balls, by even more than 100 °C, when compared to the process carried out with the tools having the roll collars wound at the angle of 720°. In most cases, the observed temperature falls on the surface, which shows that the heat is transferred from the material to the much colder tools. The temperature in the areas subjected to the highest deformation is higher than the initial value, which is due to the fact that heat is generated when the friction changes to deformation work.

In the analysis, ductile fracture was predicted by the normalized Cockcroft–Latham criterion, which is expressed as Equation (5) [17]:

(5) $$C=\int_{0}^{\varepsilon }\frac{\sigma_{1}}{\sigma_{i}}d\varepsilon$$

where: *σ*_1_—maximum principal stress, *σ_i_*—effective stress, *ε*—effective strain, *C*—Cockcroft–Latham integral value.

**Figure 12:**
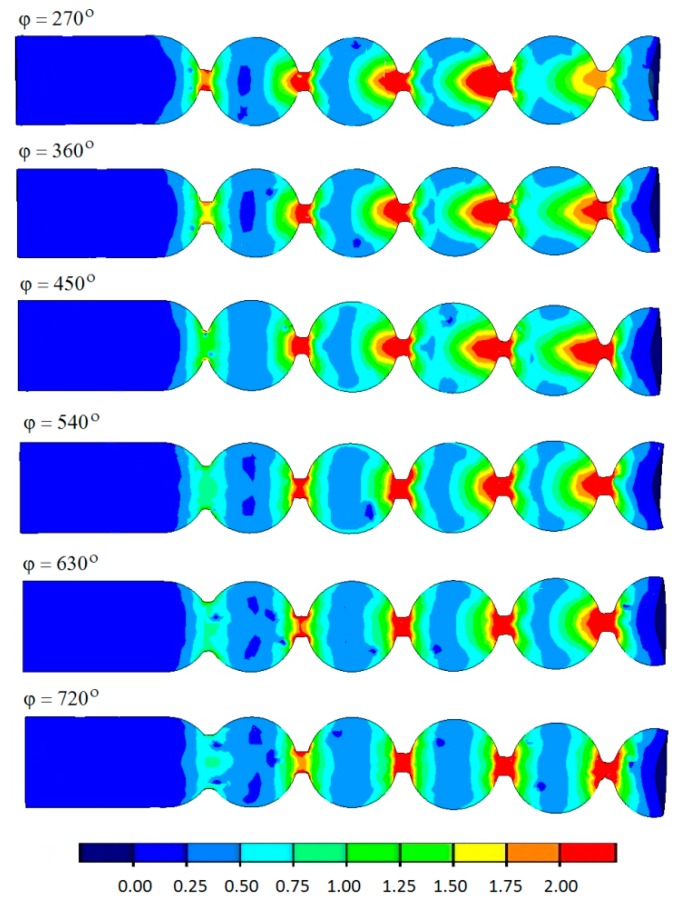
Damage (Cockcroft–Latham) in the balls produced by helical rolling.

The forming length has a significant effect on the damage function (Figure 12). It was observed that the Cockcroft–Latham integral is the highest when the tools suitable for forming balls over the shortest possible forming length (defined by the angle *φ* of 270°) are used.

With the increasing forming length, the maximum damage criterion decreases. It should also be noted that the maximum values of the Cockcroft–Latham function in the analyzed cases are higher than the limit values, and they are all located in the vicinity of the necking. Therefore, it can be expected that the material will lose cohesion in this area, and the balls will be separated. Moreover, when the balls are formed by tools with relatively short helical passes (*φ* = 270°–450°), internal cracking may occur in the produced balls, resulting from too high an increase in the strains. It should also be noted here that, in rotary forming processes, the limit values of the Cockcroft–Latham ductile fracture criterion for 100Cr6 steel are much higher than those obtained when using simple load patterns (compression and tension), and they range between 2.2 and 2.5, depending on the temperature of the material [18,19].

**Figure 13:**
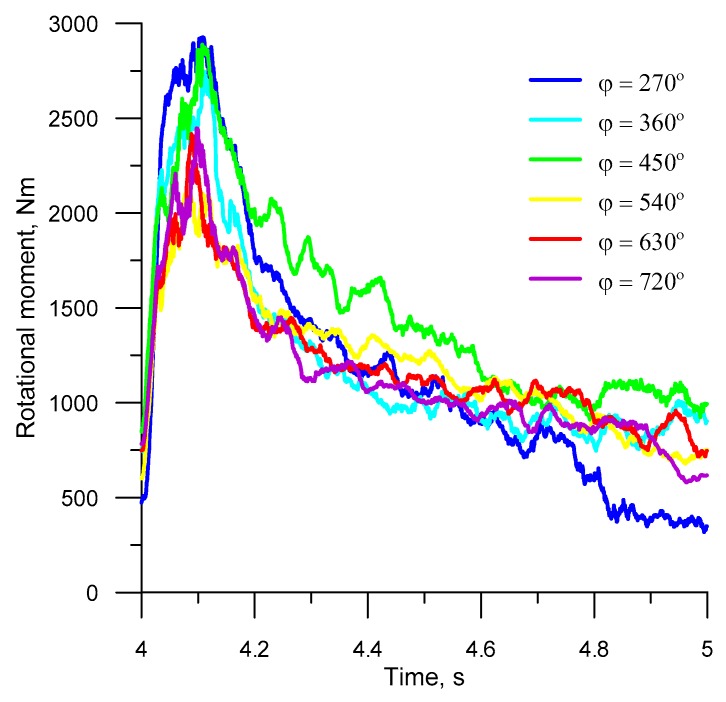
Torque during the fifth revolution of the tool.

**Figure 14:**
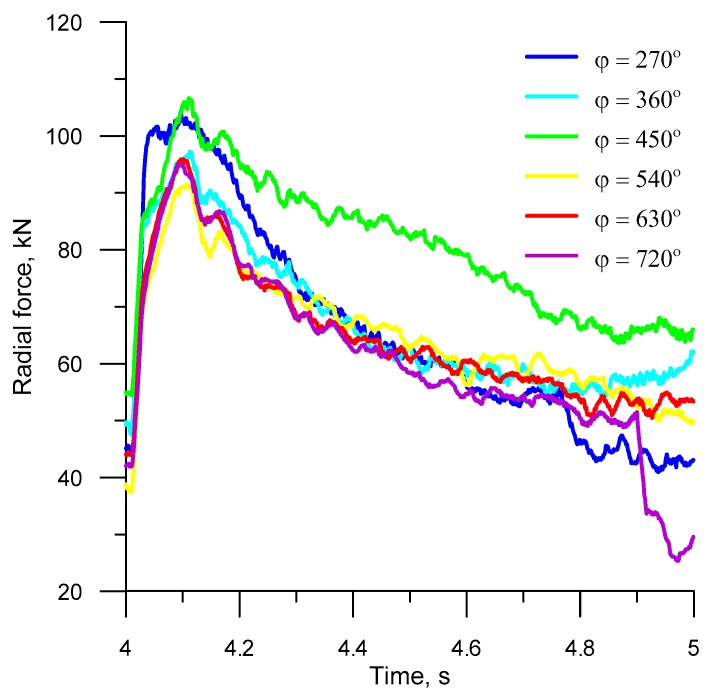
Radial force during the fifth revolution of the tool.

Figure 13 and Figure 14 show the FEM-computed torques and radial forces in the steady-state rolling (after four whole rotations of the tools), plotted for different forming lengths. As can be seen from the figures, the variations in the forces and torques are similar in all analyzed cases. What can be observed at the beginning of every full process cycle in the analyzed cases is that the forces and torques rapidly increase when the roll collars with the highest width *x* begin to cut into the material. Then, the force parameters gradually decrease by nearly 75%. After the tool has made one full revolution, the cycle is repeated.

Due to its cyclic nature, the HR process for balls is specified by high changes in the torque, which—in turn—affects the operating conditions of the rolling machine. To reduce the machine power consumption, it is recommended to use a flywheel because it accumulates energy under a lower machine load, and, thus, satisfies the instantaneously increased energy demand that occurs when the forming tools cut into the workpiece. The forming length has a significant impact on the force and torque in helical rolling. The rolling of balls with the use of tools with relatively short roll collars (*φ* = 270°–450°) generates higher force parameters (by approx. 18%), when compared to the processes in which the helical grooves are wound on the tool surface at an angle ranging between 540° and 720°. This results from a higher increase in the deformation ratio per rotation angle of the tool.

**Figure 15:**
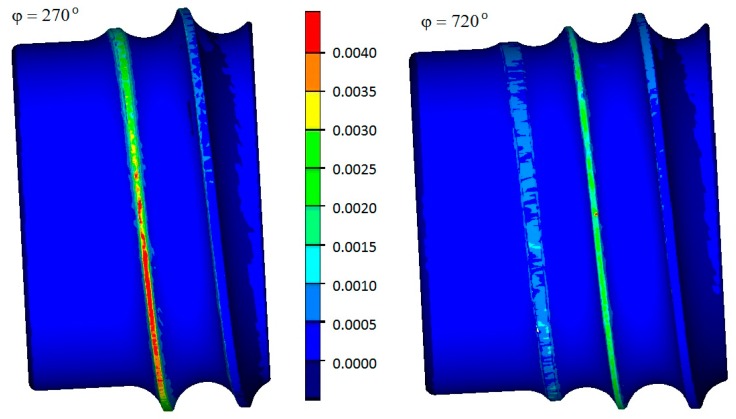
Tool wear (in mm) after five revolutions of the rolls, determined by the Archard method.

As has been demonstrated above, the forming length has a significant effect on the forces in the process of ball rolling and, thus, on the operating conditions of the tools. For this reason, the numerical simulations also involved predicting tool wear by the Archard method. The location of rapid wear zones for the tools represented by the arc from 270° to 720° is presented in Figure 15. It can be seen from the picture insofar as the increase in the forming tool length leads to a decreased wear of the tool (the wear is two times lower for the angle *φ* of 720° than for the angle *φ* of 270°). The highest wear can be observed on the surface of the roll collars in the forming area. In contrast, the effect of the roll collars located in the sizing zone is much lower, and, hence, their wear is much lower, too.

## 5. Conclusions

This study has demonstrated that both the stability of the investigated HR process and the product accuracy significantly depend on the helical tool geometry. It has been found that an increase in the forming zone length (a higher angle *φ*) has a positive effect on the accuracy of manufactured balls and the tool operation alike. Nevertheless, an excessive increase in the forming zone length (by over 720°) is not cost-effective, because it leads to a higher demand for the tool material and, as a consequence, to higher production costs of these tools.

The presented conclusions were based on the conducted research:

- complex metal forming processes, such as a HR process for balls, can be effectively modeled numerically;
- an increase in the helical tool length yields positive results because it leads to lower effective strains, the damage criterion, forces, and torques, as well as the reduced abrasion wear of tools;
- the HR process for producing balls is characterized by considerable cyclic oscillations in the values of forces and torques;
- the use of a flywheel in a rolling mill drive ensures reduced machine power consumption;
- further studies should be undertaken to specify optimum ranges of other parameters in the HR process in order to ensure the lowest possible energy and material consumption, as well as decreased tool wear.

## Figures and Tables

**Table 1:** Effect of the rotation angle *φ* in the forming zone on the tool length (according to Figure 8) in the numerical analysis.

*φ*, °	270	360	450	540	630	720
*L_k_*, mm	565.2	753.6	942	1130.4	1318.8	1507.2
*L_f_*, mm	66.9	74.3	85.4	94.7	103.3	111.5
*L*, mm	131.9	139.3	150.4	159.7	168.3	176.5
